# Aflatoxin exposure was not associated with childhood stunting: results from a birth cohort study in a resource-poor setting of Dhaka, Bangladesh

**DOI:** 10.1017/S1368980020001421

**Published:** 2021-08

**Authors:** Mustafa Mahfuz, S M Tafsir Hasan, Mohammed Ashraful Alam, Subhasish Das, Shah Mohammad Fahim, M Munirul Islam, Md Amran Gazi, Muttaquina Hossain, Patricia A Egner, John D Groopman, Tahmeed Ahmed

**Affiliations:** 1 Nutrition and Clinical Services Division, icddr,b, Dhaka 1212, Bangladesh; 2 Faculty of Medicine and Life Sciences, University of Tampere, Tampere 33520, Finland; 3 Johns Hopkins Bloomberg School of Public Health, Baltimore, MD 21205, USA

**Keywords:** Stunting, Aflatoxin B1-lysine adduct, Children, Bangladesh

## Abstract

**Objective:**

Chronic aflatoxin exposure has been associated with childhood stunting (length-for-age/height-for-age < –2 sd), while data lacks for Bangladesh, a country with substantial burden of childhood stunting. This paper examined the association between aflatoxin exposure and childhood stunting in a slum setting of Dhaka city.

**Design:**

In this MAL-ED aflatoxin birth cohort study, plasma samples were assayed for aflatoxin B1-lysine adduct (AFB1-lys) by MS at 7, 15, 24 and 36 months of age for 208, 196, 173 and 167 children to assess chronic aflatoxin exposure. Relationship between aflatoxin exposure and anthropometric measures was examined by mixed-effects logistic regression models.

**Setting and participants:**

The study was conducted in Mirpur, Dhaka, where children were followed from birth to 36 months.

**Results:**

Prevalence of stunting increased from 21 % at 7 months to 49 % at 36 months of age. Mean AFB1-lys concentrations at 7, 15, 24 and 36 months were 1·30 (range 0·09–5·79), 1·52 (range 0·06–6·35), 3·43 (range 0·15–65·60) and 3·70 (range 0·09–126·54) pg/mg albumin, respectively, and the percentage of children with detectable AFB1-lys was 10, 21, 18 and 62 %, respectively. No association was observed between aflatoxin exposure and stunting in multivariable analyses. Factors associated with childhood stunting were age, low birth weight, maternal height, stool myeloperoxidase and number of people sleeping in one room.

**Conclusions:**

A relatively lower exposure to aflatoxin may not influence the linear growth of children. This finding indicates a threshold level of exposure for linear growth deficit and further investigation in other areas where higher concentrations of aflatoxin exposure exist.

Stunting (length-for-age *z*-score (LAZ)/height-for-age *z*-score (HAZ) < –2 sd of WHO growth standards) or chronic undernutrition is considered the most pervasive form of childhood malnutrition affecting 150 million children under the age of 5 years globally^(1,2)^. Recently published papers from the multi-country MAL-ED birth cohort study demonstrated that low birth weight, low maternal height, higher burden of non-diarrhoeal enteropathogens in stool samples, lower socioeconomic status (SES) and inadequate protein content in the diet are the predictors of childhood stunting at 24 months of age^(3,4)^. Additionally, poor water, sanitation and hygiene (WASH) behaviour as well as exposure to environmental toxins are the other known risk factors for childhood stunting^(5–9)^. Data from Africa showed that environmental toxin, particularly dietary aflatoxin exposure, is associated with linear growth faltering in children^(6–11)^. Aflatoxins are secondary metabolites of *Aspergillus* species, which are known to contaminate most of the African staples^(12)^. With an estimated prevalence of 36 %, Bangladesh is considered to be a country with the highest burden of childhood stunting among the children <5 years of age^(13)^. Moreover, the prevalence of linear growth retardation is as high as 50 % in slum areas of the country^(14)^. Despite the limited availability of exposure data, it can be assumed that the hot and humid climate of Bangladesh is conducive for fungal growth and subsequent toxin production^(6,15)^. Aflatoxin exposure has been well documented in food commodities and human studies conducted in Bangladesh^(16–18)^. Recent data showed that 62 % of children in Dhaka slums were exposed to aflatoxin at 36 months of age, and the exposure was found to be associated with the end of rainy season and introduction of family food^(19)^.

Aflatoxin is the most known and extensively studied mycotoxin for its role in the pathogenesis of liver cancer^(20)^. A number of animal studies and few human studies provided evidence for its association with fetal growth retardation, low birth weight and childhood stunting^(20)^. The possible mechanisms include its interference in the metabolism of carbohydrates, protein and fatty acid synthesis, damage to enterocytes leading to poor nutrient absorption and utilisation, Zn deficiency and systemic immune activation^(9,10,21,22)^. Aflatoxin exposure may also interrupt the insulin-like growth factor (IGF) pathway, which has been demonstrated in a study conducted in Kenya. The study results revealed an inverse relationship between aflatoxin-albumin adduct (AF-alb) and IGF1 concentrations, and showed that 16 % of child height deficits can be explained by low IGF1 levels^(23)^. A longitudinal study conducted in Benin and Togo provided explicit evidence on a dose–response relationship between aflatoxin exposures measured by AF-alb and HAZ of children, and ignited the public health community to further explore this relationship through a cascade of researches^(9,10)^. On the other hand, childhood stunting is believed to be associated with environmental enteropathy, a poorly understood chronic inflammatory condition that mainly affects the small intestine of an individual^(24,25)^. Environmental enteropathy is characterised by alteration of small intestinal structure, intestinal inflammation and increased gut permeability, owing to leakiness of intestine. Persistent dietary exposure to aflatoxin during childhood may induce enterocyte damage and partially explain the gut ‘leakiness’, which impairs efficient absorption and harvesting of nutrients from the diet, and ultimately results in malabsorption of essential nutrients^(20)^. Therefore, biomarkers of environmental enteropathy need to be considered to examine the association between aflatoxin exposure and childhood linear growth faltering.

This current analysis uses data from the MAL-ED aflatoxin study, a companion study of MAL-ED birth cohort study conducted in Dhaka, Bangladesh. In MAL-ED birth cohort, children were followed longitudinally from birth to beyond 36 months of age, and data were collected systematically on most of the variables associated with childhood growth, including sociodemography; maternal information; child feeding practices, including exclusive breastfeeding days; hand washing practice; treatment of drinking water; and presence of hygienic toilets^(26)^. Moreover, data on different biomarkers of environmental enteropathy are also available in children at different time-points^(4,26,27)^. On the other hand, the MAL-ED aflatoxin study utilised the blood samples collected from the children of MAL-ED birth cohort study at different time-points, and performed assays using MS to detect aflatoxin B1-lysine (AFB1-lys), a marker of chronic aflatoxin exposure. Most of the available literatures, particularly from birth cohort studies, examined the risk factors of stunting at 24 months of age. However, there remains paucity of data on the determinants of stunting at 36 months of age. Given the high burden of childhood stunting in the slum areas of Dhaka, this birth cohort study examined the association of aflatoxin exposure and childhood stunting from 7 to 36 months of age.

## Materials and methods

### Study design and participants

The study site was located in the Bauniabadh slum area of Mirpur, Dhaka, which is a densely populated slum settlement inhabited by people with low SES with sub-optimal sanitary conditions. Detailed information about the study site, geography and sociodemography has been published elsewhere^(4,26,27)^. In this birth cohort study, newborns were enrolled within 17 d of birth with an average age at enrolment of 3·4 d, and followed longitudinally beyond 36 months of age. Well-defined inclusion and exclusion criteria were used to enrol the participants. The inclusion criteria include apparently healthy newborn within 17 d of birth, parents had no plan to migrate out in the next 6 months, and caregiver agreed to be visited at home by research staff twice weekly. The exclusion criteria include the family having a plan to move outside the study area, newborn baby with very low birth weight (<1·5 kg), maternal age <16 years, multiple pregnancy, another child from the same family enrolled in the study, severe disease requiring hospitalisation and chronic disease or congenital anomalies^(27)^. Participant enrolment started in February 2010. To cover seasonal variations, enrolment continued till February 2012 with an average enrolment of ten participants per month. Among the 229 enrolled newborns with complete data at baseline, 212 children completed 24-month follow-up, and 196 of them were followed through 36 months of age. Blood samples were collected at the age of 7, 15, 24 and 36 months. Among the children with available plasma samples, 208 of them who provided consent to use their samples for aflatoxin assays were enrolled in MAL-ED aflatoxin study^(19)^.

### Data collection

During enrolment, date of birth and birth weight of each child were recorded, and data relating to breastfeeding status, including initiation of breastfeeding, baseline sociodemographic information and anthropometric measurements of children and mothers were collected^(26–28)^. Through twice-weekly home visits by research staff, intensive dietary and morbidity data were collected^(29)^. Detailed information regarding the methodology was published previously^(27,28)^. Anthropometric data were collected each month, and data on WASH behaviour, assets, income and food security were collected every 6 months^(19,27)^.

### Biological sample collection

Through longitudinal visits, blood and stool samples were collected using a standard MAL-ED protocol. Blood samples were collected at 7, 15, 24 and 36 months of age, and plasma was obtained by centrifugation of blood samples. Monthly stool samples were collected without a fixative, aliquoted and stored at –70°C^(19)^.

### Aflatoxin plasma biomarker assay

AFB1-lys is a well-established and sensitive biomarker of long-term aflatoxin exposure. We performed this assay at Groopman’s Laboratory at Johns Hopkins University, using previously published methods^(30)^. We used isotope dilution MS to detect the concentration of plasma samples. In short, 200 µl of plasma was combined and vortexed with an internal standard (10 µl × 0·1 ng AFB 1-D4-lys per millilitre) and pronase (Millipore Corp.; Catalogue no. 537088 – 100 µm) and incubated at 37°C for 18 h. Samples were then passed across a solid-phase extraction column (Waters Oasis^®^ MAX Cartridge; 1 cc/30 mg; Catalogue no. 186000366). The eluent was analysed using UPLC with an MS detection system. The AFB1-lys molecular ion (*m*/*z* 457·2) fragmented to yield a daughter ion at *m*/*z* 394·1, and the parent ion of internal standard ((M + H)^+^, *m*/*z* 461·3) fragmented to yield a daughter ion at *m*/*z* 398·2. The limit of detection for this method was 0·5 pg AFB1-lys/mg albumin, and three quality control (QC) samples ran daily^(19)^.

### Biomarkers of environmental enteropathy

The biomarkers of environmental enteropathy, including α1 antitrypsin (AAT), neopterin (NEO), myeloperoxidase (MPO) and regenerating protein family member 1β (Reg1B), were measured from non-diarrhoeal stool samples. All assays were performed at icddr,b, Dhaka. AAT (Biovendor), NEO (GenWay Biotech), MPO (Alpco) and Reg1B (TechLab) were measured in stool samples using commercially available ELISA kits and following the manufacturers’ instructions. The overall methodology of biomarker assays has already been described^(31)^.

### Anthropometry

The outcome of this analysis was linear growth of children measured in LAZ or HAZ. LAZ (0–24 months)/HAZ (36 months) was calculated from the length/height and weight of children collected during each monthly visit to the study field office. Anthropometry was conducted by two trained research staff following standard operating procedures. Length was measured using commercial measuring boards (Seca Infantometer; model no. 417); height was measured with Seca 213 portable stadiometer; and weight was measured with minimum clothing using Seca 354 Dual-Purpose Baby Scale. Anthropometric indices were calculated following WHO growth standards^(32)^. All the instruments were calibrated daily with standard weights and a measuring rod. Details of anthropometry, equipment and data on QC were published elsewhere^(4,27)^.

### Variable selection for analysis

Aflatoxin exposure is the explanatory variable of interest, which was measured at the ages of 7, 15, 24 and 36 months. Therefore, in addition to baseline information, data on covariates were considered, which were collected only at these particular time-points. The outcome variables of this analysis were LAZ/HAZ and stunting status at the ages of 7, 15, 24 and 36 months. In order to select explanatory variables, we considered the variables included in the MAL-ED study’s pooled analyses to explore the predictors of stunting, which followed a modified version of the UNICEF malnutrition conceptual hierarchical framework and also used the maternal and household factors and childhood environmental exposures^(4)^. In addition, we considered variables used in other reported contemporary studies to explore the risk factors of childhood stunting. Name and availability of different variables across different time-points in this longitudinal study are described in online supplementary material, Supplemental Table 1.

### Definitions

*Asset index:* A household asset index was constructed using household asset data obtained from the SES questionnaire. From these asset-related dichotomous variables, a common factor score for each household was generated using polychoric principal components analysis in STATA software. After ranking by their score, we divided the first principal component score into quintiles to create five categories where the first category represents poorest household, and the fifth category represents wealthiest household.

Improved toilet was defined as per WHO guidelines: presence of flush latrine to piped sewer system, septic tank, pit latrine; ventilated improved pit latrine; pit latrine with slab; or composting toilet^(33)^.

Household food security status was categorised using the Household Food Insecurity Access Scale (HFIAS) developed by Food and Nutrition Technical Assistance project^(34)^.

### Statistical analysis

We examined the distribution of variables and characterised their distributions using histograms, means and standard deviations, or frequency tables as appropriate. Continuous variables that were not normally distributed were characterised by median and interquartile range. A descriptive statistics was performed to present the characteristics of study participants. To investigate the independent relationship between aflatoxin exposure and LAZ/HAZ, we fitted a mixed-effect multiple linear regression model specifying a random effect at the child level to account for within-child correlations. To account for multiple measurements per child, we calculated robust standard errors. Similarly, we investigated the independent relationship between aflatoxin exposure and stunting by fitting a mixed-effect multiple logistic regression model. Multicollinearity was checked by calculating the variance inflation factor (VIF) in a series of single-level linear regression models. The variables in the final models had a VIF ≈ 2. The strength of association was measured by OR with 95 % CI. For multivariable model building, all the time-independent covariates, and the variables collected at four time-points (7, 15, 24 and 36 months) among the time-varying covariates, were considered. Statistical significance was set at *P* < 0·05. We performed all the statistical analyses using Stata/PC (StataCorp, version 15·1).

## Results

A total of 228 children were enrolled in this study, and 196 were followed till 36 months of age. Baseline data showed that 52 % of the children were female; mean (sd) birth weight was 2·8 (0·4) kg, and 22 % of the children were born with low birth weight (<2·5 kg). Prevalence of stunting (LAZ/HAZ < –2 sd) was 22 % at birth, and median duration of exclusive breastfeeding was 107 (interquartile range 54, 155) d. The mean maternal height was 149 (sd 5) cm, and the average duration of formal education of the mothers was 5 years. Monthly family income of the enrolled children was $US 101, and the asset index showed that 21 % of the children were from the poorest families, and 18·7 % of the children came from poor families. Seventy-four percentage of the children came from food-secured households, and 7·7 % of the children came from households with severe food insecurity. Seventy-three percentage of the mothers washed their hands after helping the child defecate; 20 % of them washed their hands before food preparation; and 76 % washed their hands after using the toilet. Seventy-five percentage of the children had access to improved toilet, and 61 % of their families treated water by any means before drinking (Table 1).

**Table 1:** Baseline characteristics of children enrolled in this study

Characteristic	Mean or *n*	sd or % or IQR
Sample size	229	–
Sample size of children included in the final analysis	205	–
Female sex, *n* and %	119	51·9
Birth weight (kg), mean and sd	2·81	0·41
Low birth weight (<2·5 kg), *n* and %	50	21·8
Length-for-age *z*-score < –2 at birth (%)	37	16·2
Maternal height (cm), mean and sd	148·8	5·12
Duration of exclusive breastfeeding (d), median and IQR	107	54, 155
Maternal education (years), median and IQR	5	2,7
Monthly family income (USD), median and IQR	101·3	75·9, 126·6
HFIAS category		
Food secure	171	74·5
Mild insecure	14	6·3
Moderate insecure	26	11·5
Severe insecure	18	7·7
Asset index*, *n* and %		
Poorest	49	21·4
Poor	43	18·7
Middle	48	20·9
Wealthy	46	20·3
Wealthiest	43	18·7
Number of people sleeping in one room, mean and sd	3·65	1·11
Hand-washing after helping child defecate, *n* and %	166	72·6
Hand-washing before preparing food, *n* and %	45	19·7
Hand-washing after using the toilet, *n* and %	174	75·9
Improved toilet†, *n* and %	173	75·5
Drink treated water, *n* and %	139	60·6

IQR, interquartile range; HFIAS, Household Food Insecurity Access Scale.

* Asset index: The household asset index was constructed using household asset data obtained from the Socioeconomic Status questionnaire. From these asset-related dichotomous variables, a common factor score for each household was generated using polychoric principal components analysis in STATA software. After ranking by their score, we divided first principal component score into quintiles to create five categories where the first category represents the poorest household and the fifth category represents the wealthiest household.

† Improved toilet was defined as per WHO guidelines: presence of flush latrine to piped sewer system, septic tank, pit latrine; ventilated improved pit latrine; pit latrine with slab; or composting toilet.

To detect aflatoxin exposure among the children, AFB1-lys assays were performed using the blood samples collected at the ages of 7, 15, 24 and 36 months. Assay results were available for 208, 196, 173 and 167 children, and aflatoxin was detected in 10, 20, 17 and 62 % of samples, respectively, at those time-points. The mean LAZ values were –1·29, –1·80, –2·03 and –1·99 at the ages of 7, 15, 24, and 36 months, respectively. The prevalence of stunting was 21 % at 7 months, 41 % at 15 months, 49 % at 24 months and 49 % at 36 months of age. Details on aflatoxin concentrations, LAZ/HAZ values, Hb concentrations and concentrations of enteropathy biomarkers, including stool MPO, NEO, AAT and Reg1B measured at 7, 15, 24 and 60 months, are reported in Table 2.

**Table 2:** Growth and laboratory assay results of the children between 7 and 36 months

	Month 7		Month 15		Month 24		Month 36	
	Mean/*n*/median	sd/IQR/%	Mean/*n*/median	sd/IQR/%	Mean/*n*/median	sd/IQR/%	Mean/*n*/median	sd/IQR/%
Children with available data, *n*	208	–	196	–	173	–	167	–
LAZ/HAZ, mean and sd	–1·29	0·96	–1·80	0·93	–2·03	0·9	–1·99	0·8
Stunting prevalence, *n* and %	43	20·7	81	41·2	86	49·7	82	49·1
Aflatoxin detected, *n* and %	21	10·1	40	20·4	30	17·3	102	61·2
Concentration of AFB1-lys (pg/mg)								
Mean and sd	1·30	1·5	1·52	1·5	3·43	11·8	3·70	12·9
Median and IQR	0·83	0·3, 1·6	1·14	0·5, 2·0	0·95	0·6, 1·2	1·17	0·7, 2·9
MPO (mcg/ml), median and IQR	3·40	1·57, 6·83	4·34	2·21, 7·73	2·02	1·29, 2·88	2·75	1·08, 4·78
NEO (mcmol/l), median and IQR	1·36	0·51, 2·76	1·59	0·70, 2·78	0·67	0·27, 1·38	0·37	0·21, 0·73
AAT (mg/g), median and IQR	0·42	0·2, 0·8	0·38	0·19, 0·71	0·36	0·2, 0·7	0·28	0·15, 0·5
Reg1B (µg/ml), median and IQR	23·6	8·8, 52·5	76·9	42·9, 132·4	52·9	9·1, 95·7	21·1	6·4, 67·7
Hb (mmol/L), mean and sd	6·9	0·8	7·1	0·9	7·3	0·9	7·6	0·8

LAZ, length-for-age *z*-score; HAZ, height-for-age *z*-score; IQR, interquartile range; AFB1-lys, aflatoxin B1-lysine adduct; MPO, myeloperoxidase; NEO, neopterin; AAT, α1 antitrypsin.

### *Association between aflatoxin exposure and* length-for-age *z*-score/height-for-age *z*-score *of children*

To examine the association between aflatoxin exposure and LAZ/HAZ in mixed-effect linear regression models, unadjusted analyses showed that the detection of aflatoxin was inversely associated with LAZ/HAZ (–0·19, 95 % CI –0·28, –0·11, *P* < 0·05). Similarly, compared with children at 7 months of age, LAZ/HAZ values were lower in 15 months (–0·55, 95 % CI –0·63, –0·48, *P* < 0·05), 24 months (–0·77, 95 % CI –0·86, –0·67, *P* < 0·05) and 36 months (–0·71, 95 % CI –0·81, –0·62, *P* < 0·05) of age (Table 3). Among other explanatory variables, low birth weight and the number of people sleeping in one room were also inversely associated with LAZ/HAZ of children over the period from 7 to 36 months in bivariate analyses. On the other hand, maternal height, treatment of drinking water, MPO concentration in stool and asset index (wealthier compared with children from poorest households) were positively associated with LAZ/HAZ. The multivariable model did not show any association between aflatoxin exposure and LAZ/HAZ (0·03, 95 % CI –0·06, 0·11, *P* = 0·54). The multivariable model showed that age was inversely associated with LAZ/HAZ. Compared with children aged 7 months, LAZ/HAZ values were reduced in 15 months (–0·58, 95 % CI –0·66, –0·5, *P* < 0·05), 24 months (–0·77, 95 % CI –0·87, –0·67, *P* < 0·05) and 36 months (–0·74, 95 % CI –0·84, –0·63, *P* < 0·05) of age. Other factors positively associated with LAZ/HAZ in adjusted analyses were female sex (0·22, 95 % CI 0·2, 0·42, *P* < 0·05), maternal height (0·04, 95 % CI 0·02, 0·07, *P* < 0·05), access to improved toilet (0·26, 95 % CI 0·4, 0·49, *P* < 0·05) and MPO concentration (0·004, 95 % CI 0·0004, 0·008, *P* < 0·05) in stool. Factors inversely associated with LAZ/HAZ were low birth weight (–0·69, 95 % CI –0·94, –0·44, *P* < 0·05) and number of people sleeping in one room (–0·14, 95 % CI –0·22, –0·05, *P* < 0·05) (Table 3).

**Table 3:** Bivariate and multivariable analyses of factors associated with length-for-age/height-for-age over 7–36 months of age

	Unadjusted			Adjusted		
	Coefficient	95 % CI		Coefficient	95 % CI	
Aflatoxin detected	–0·19*	–0·28	–0·11	0·03	–0·06	0·11
Target months (reference: 7 months)						
15 months	–0·55*	–0·63	–0·48	–0·58*	–0·66	–0·5
24 months	–0·77*	–0·86	–0·67	–0·77*	–0·87	–0·67
36 months	–0·71*	–0·81	–0·62	–0·74*	–0·84	–0·63
Female sex	0·13	–0·09	0·37	0·22*	0·02	0·42
Low birth weight	–0·66*	–0·94	–0·39	–0·69*	–0·94	–0·44
Maternal height	0·06*	0·04	0·08	0·04*	0·02	0·07
Improved toilet	0·25	–0·02	0·52	0·26*	0·04	0·49
Treat drinking water	0·38*	0·16	0·61	0·21	–0·002	0·41
Myeloperoxidase (mcg/ml)	0·01*	0·004	0·02	0·004*	0·0004	0·008
Number of people sleeping in one room	–0·18*	–0·29	–0·08	–0·14*	–0·22	–0·05
Asset index (reference: poorest)						
Poor	0·54*	0·16	0·92	0·25	–0·13	0·63
Middle	0·66*	0·27	1·04	0·29	–0·08	0·67
Wealthier	0·49*	0·13	0·85	0·13	–0·20	0·47
Wealthiest	0·97*	0·59	1·34	0·29	–0·10	0·69

* Statistical significance at *P* < 0·05.

### Association of aflatoxin exposure with stunting

We also examined the association of aflatoxin exposure and stunting. Results of both unadjusted and adjusted models are presented in Table 4. In unadjusted analyses, the detection of aflatoxin, age, low birth weight, maternal height, treatment of drinking water, MPO concentration in stool, number of people sleeping in one room and asset index were associated with stunting. Although the unadjusted analysis showed that the detection of aflatoxin was associated with increased odds of stunting (OR 2·2, 95 % CI 1·34, 3·67, *P* < 0·05), we did not find any association between these two in the multivariable model (adjusted OR (AOR) 0·9, 95 % CI 0·4, 1·9, *P* = 0·82). In the adjusted analyses, age was also associated with stunting as there were higher odds of being stunted at 15 months (AOR 24·4, 95 % CI 8·2, 72·4, *P* < 0·05), 24 months (AOR 38·5, 95 % CI 9·8, 151·3, *P* < 0·05) and 36 months (AOR 39·8, 95 % CI 10·2, 155·7, *P* < 0·05) of age compared with 7 months. Being female had 84 % lower odds of being stunted compared with being male (AOR 0·16, 95 % CI 0·04, 0·07, *P* < 0·05); low-birth-weight children had thirty-six times higher odds of being stunted compared with normal-birth-weight children (AOR 36·3, 95 % CI 5·29, 249·11, *P* < 0·05); and every unit increase in maternal height was associated with 21 % lower odds of being stunted (AOR 0·79, 95 % CI 0·67, 0·93, *P* < 0·05). Moreover, MPO concentration in stool was inversely associated with stunting, and every unit increase in the number of people sleeping in one room had 2·2 times higher odds of a child being stunted (AOR 2·21, 95 % CI 1·14, 4·27, *P* < 0·05) (Table 4). We did not find an association between asset status and stunting.

**Table 4:** Factors associated with stunting at 7–36 months of age: results from unadjusted and adjusted regression models

	Unadjusted			Adjusted		
	OR	95 % CI		OR	95 % CI	
Aflatoxin detected	2·22*	1·34	3·67	0·91	0·43	1·94
Target months (reference: 7 months)						
15 months	17·54*	6·73	45·69	24·35*	8·19	72·37
24 months	35·71*	10·81	117·97	38·51*	9·80	151·29
36 months	38·38*	11·76	125·28	39·78*	10·16	155·71
Female sex	0·40	0·14	1·12	0·16*	0·04	0·70
Low birth weight	8·94*	2·71	29·48	36·32*	5·29	249·11
Maternal height	0·79*	0·72	0·88	0·79*	0·67	0·93
Improved toilet	0·36	0·11	1·22	0·23	0·04	1·19
Treat drinking water	0·23*	0·08	0·63	0·29	0·07	1·26
Myeloperoxidase (mcg/ml)	0·93*	0·89	0·97	0·92*	0·87	0·97
Number of people sleeping in one room	1·83*	1·14	2·93	2·21*	1·14	4·27
Asset index (reference: poorest)						
Poor	0·20*	0·04	0·99	0·32	0·03	4·06
Middle	0·08*	0·01	0·42	0·11	0·01	1·43
Wealthier	0·18*	0·04	0·84	0·49	0·05	4·92
Wealthiest	0·04*	0·007	0·23	0·30	0·02	5·16

* Statistical significance at *P* < 0·05.

## Discussion

The primary objective of this study was to examine the association of chronic aflatoxin exposure with childhood stunting from 7 to 36 months of age. We did not find any independent association between chronic aflatoxin exposure and childhood stunting after performing longitudinal data analysis in the multivariable model. Although aflatoxin exposure was found to be inversely associated with childhood stunting in the unadjusted analysis, the association became non-significant after adjusting for age. Since both aflatoxin and LAZ/HAZ data were collected at the ages of 7, 15, 24 and 36 months, and the rate of exposure to aflatoxin as well as the prevalence of stunting significantly increased with age (Table 2), the crude association between aflatoxin exposure and stunting more likely represented an association between increase in age and stunting. The absence of a relationship between aflatoxin exposure and LAZ/HAZ or stunting became more evident when we examined this relationship by fitting multiple (linear and logistic) regression models separately for each time-point (see online supplementary material, Supplemental Tables 1 and 2).

Our finding was similar to recently published MAL-ED consortium studies conducted in Tanzania and Nepal, where they did not find any relationship between aflatoxin exposure and linear growth of children measured in LAZ/HAZ^(35,36)^. For the detection of aflatoxin exposure, we applied the same method (MS) used in Tanzania and Nepal, and assays were performed in the same laboratory^(35,36)^. In our study, 62 % of children had a detectable concentration of AFB1-lys in plasma at 36 months. The detection was much lower in samples collected at the age of 7 months (10 %), 15 months (20 %) and 24 months (17 %). In the Tanzanian study, Chen *et al*.^(35)^ had performed AFB1-lys assays only at 24 months of age where 72 % of children had a detectable level of AFB1-lys. The Nepal study measured aflatoxin at three time-points, 15, 24 and 36 months, with an average 91 % of detectable AFB1-lys concentrations in plasma. Our sample size was much larger than both the studies. Compared with the total number of children with available aflatoxin assay results in our study (7 months: *n* 208; 15 months: *n* 196; 24 months: *n* 173; 36 months: *n* 167), the number of children with available aflatoxin exposure data in Tanzania (24 months: *n* 60) and Nepal (15 months: *n* 77; 24 months: *n* 85; 36 months: *n* 85) were much lower^(35,36)^.

All three studies observed a lower concentration of AFB1-lys in plasma samples, which was 5 pg/mg or less compared with previous studies (median > 30 pg/mg)^(9,10,35,36)^. A lower concentration of AFB1-lys in detected plasma samples could be the main reason behind the non-association with the linear growth of children. The detectable concentrations were similar in Tanzania and Nepal studies. In Tanzania, the mean concentration of AFB1-lys at 24 months was 5·1 (range 0·28–25·1) pg/mg albumin, and this was 3·62 (range 0·58–22·7) pg/mg of albumin in Nepal^(35,36)^. In our study, the mean concentrations were 3·43 (range 0·15–65·6) pg/mg at 24 months and 3·70 (range 0·09–126·5) pg/mg at 36 months.

Recently, another cohort study conducted in Tanzania also found no association between LAZ and aflatoxin exposure^(37)^. They enrolled 166 children between the ages of 8 and 20 months and followed them for 12 months. ELISA was used to detect AFB1-alb, and the assays were done at three time-points: at baseline, at 6 months and at 12 months. The detected mean concentrations of AFB1-alb were 4·7, 12·9 and 23·5 pg/mg, respectively, at the three time-points^(37)^. Both Nepal and Tanzania studies used a scaling factor of 2·6 to adjust for methods between ELISA and MS based on previously published data, and found no difference with the concentration observed by Shirima *et al*.^(37)^. It can be noted that ELISA and LC-MS/MS correlated strongly with each other, but ELISA usually quantified AFB-alb by a factor of 2·6 higher than LC-MS/MS as observed in previous studies^(38)^. Therefore, this 2·6 scaling factor was used previously to make the results comparable between ELISA and LC-MS/MS. Recently in the north of Bangladesh, a high level of AFB1-lys was detected by MS among 61 newborn infants and the same children at the age of 2 years^(20)^. The detected median concentration of AFB1-lys was 27·41 (range 3·88–81·44) pg/mg at birth and 13·79 (range 3·88–81·44) pg/mg at 2 years^(18)^. Unfortunately, no data on its association with child growth is available till date.

In the recent past, several epidemiological studies conducted in Africa and the Middle East showed an association between aflatoxin exposure and growth deficits in children^(10,11,39–42)^. The study conducted in Benin showed a significant inverse relationship between concentrations of AF-alb and different quartiles of LAZ of children. In contrast to our current research and other studies conducted in Nepal and Tanzania, the detected AFB1-alb concentration in Benin was as high as 100 pg/mg in children belonging to the lowest LAZ quartile^(9,10)^. Even after using the scaling factor of 2·6 in Benin and Togo study, the mean AFB1-alb concentrations became 32·8 pg/mg of albumin, a much higher concentration than the recent studies that found no association with growth. Therefore, we also concurred with the Nepal and Tanzania studies that exposure to a comparatively lower concentration of aflatoxin may not affect the linear growth of children^(35,36)^. It is possible that a threshold exists for intake or exposure level, and a long-standing aflatoxin exposure above this threshold will cause growth deficits as seen in previous studies conducted in Benin and Togo.

Dietary habits may play an important role in the exposure of aflatoxin to children. Usually, children in Bangladesh do not consume maize and peanut as staples, like those in African countries^(19)^. Previously, using a 24-h recall method to calculate dietary intakes in the same population, we observed that the consumption of any sweet foods such as biscuits, pastries or cakes was associated with the detection of aflatoxin in plasma (AOR 2·17, 95 % CI 1·27, 3·70, *P* < 0·05)^(19)^. We did not find any association between the consumption of grains (rice, bread, porridges, noodles, etc.) and aflatoxin exposure. However, we observed that the introduction of family food as reflected by a cessation of breastfeeding was associated with the detection of AFB1-lys in plasma^(19)^. Similar to the current study, the rate of growth impairment increased with age in some African countries where they also found that cessation of breastfeeding and introduction of family food were associated with aflatoxin exposure. However, this exposure was mainly due to the consumption of weaning food prepared from maize and peanut^(9,10,43)^.

In order to explore the true association of aflatoxin exposure with childhood stunting and LAZ/HAZ, we adjusted for other important confounding variables in the multivariable models. We observed that age, sex, low birth weight, maternal height, crowding as represented by the number of people sleeping in one room, presence of improved toilet and MPO concentration in stool were significantly associated with linear growth and stunting between the ages of 7 and 36 months. Low birth weight and short maternal stature are already considered as the most important predictors of childhood stunting^(4)^. The number of people sleeping in one room represents crowding, which also is related to poor hygiene and pathogen transmission. MPO is a biomarker of enteropathy, which was also found to be associated with linear growth^(44)^. It can be noted that a couple of papers used the MAL-ED data to explore the factors associated with childhood stunting^(4,45)^. Our findings are consistent with both the papers except that they did not examine the effect of aflatoxin exposure, and their study population was limited to 24 months of age.

So far, no data has been published from this current population to examine the factors associated with childhood stunting where children were followed until 36 months of age. Moreover, this is the first report from Bangladesh where the association of aflatoxin exposure and stunting is examined, and very few global studies with cohort data performed a robust MS assessment at four time-points with a reasonably large sample size. Among the other three recent studies, the Nepal study assayed AFB1-lys at three time-points (15, 24 and 36 months) with a small sample size (*n* 85); the Tanzania study performed aflatoxin assays at a single time-point (24 months) with a small sample (*n* 60); and the second Tanzania study performed assaying using ELISA^(35–37)^.

The findings of this study corroborate with recent studies that questioned the existing evidence linking aflatoxins with stunting. Earlier observational studies overlooked certain confounding factors, including SES^(9,10)^. Children in poorer households were often fed diets deficient in micro- or macronutrients, and suffer from frequent infections, both of which contribute to growth retardation^(3)^. Poverty is also associated with aflatoxin exposure, so, if not adequately controlled for, the association between aflatoxin/mycotoxin and stunting might be overestimated. A very recent RCT in Kenya also observed that providing aflatoxin-free maize can substantially reduce serum AFB1-lys, but it had no effect in improving linear growth faltering in children^(46)^. Therefore, it is possible that a true association between aflatoxin exposure and childhood growth faltering might not exist at all. Recently, one systematic review examined the association of mycotoxin exposure and child growth and other outcomes^(47)^. They have reviewed fifty articles and examined the evidence on aflatoxin and fumonisin exposure on child growth outcomes. They found that the results were inconsistent and inconclusive, and the evidence was considered very low due to study design and methodological issues^(47)^.

This study has several limitations. First, aflatoxin exposure data were missing at several time-points. This was due to the unavailability of biospecimens due to some participants not turning out for blood collection. The weaning period is the most vulnerable time of aflatoxin exposure when a child is gradually exposed to family foods. Since we do not have aflatoxin exposure data at 2–3 months of age, we could not relate this to 7-month exposure data when weaning would have already initiated. The unavailability of aflatoxin concentration in common food is also a limiting factor. The absence of other important predictors, such as pathogen burden data after 24 months of age, is another limitation. Moreover, exposure to other mycotoxins, for example, fumonisin, which has a role in growth faltering^(47)^, was not measured in this study.

Stunting at an early age is associated with an increased risk of childhood death, diseases and poor cognitive outcomes^(48,49)^. Despite substantial improvement, childhood stunting is pervasive in Bangladesh, particularly in slum settings. About half of children under the age of 5 years living in around 15 000 slums across Bangladesh are stunted^(14,50)^. Our analysis identified different modifiable factors associated with stunting among children aged ≤36 months, which are consistent with previously reported predictors of stunting under the age of 2 years. Improvement of birth weight and maternal height calls for improving maternal and adolescent nutrition on a large scale. Moreover, the improvement of SES of slum dwellers, provision of better living conditions without much crowding, and proper sanitation and hygiene to prevent environmental enteropathy need to be ensured through proper policy and planning using a multi-departmental approach at the national level.

Similar to recent studies conducted in Tanzania and Nepal, we found that exposure to a low level of aflatoxin did not affect the linear growth of children. However, there can be a threshold level of the toxin to demonstrate any effect on linear growth during early years of life. We need to explore further in other rural settings where exposure to a high level of aflatoxin may be more likely among children and adults. Furthermore, well-designed studies are required to detect aflatoxin in food commodities and determine the threshold value of the toxin to possibly cause an adverse impact on health.

## Supplementary material

For supplementary material accompanying this paper visit https://doi.org/10.1017/S1368980020001421. click here to view supplementary material
