# Supplementary material for: Aflatoxin exposure was not associated with childhood stunting: results from a birth cohort study in a resource-poor setting of Dhaka, Bangladesh
Source: Public Health Nutr. 2020 Jul 3;24(11):3361–70. doi: 10.1017/S1368980020001421 (PMC8314919; doi:10.1017/S1368980020001421)
Supplement: Supplementary file 1 [file S1368980020001421sup001.docx]

**Supplementary table 1**: Availability of variables at different time points in this longitudinal study

| Factors | Variables | Baseline | 7 month | 15 months | 24 months | 36 months |
| --- | --- | --- | --- | --- | --- | --- |
| Outcome | LAZ  Stunting |  | + | + | + | + |
| Explanatory variables | |  |  |  |  |  |
| Explanatory variable of interest | Aflatoxin exposure |  | + | + | + | + |
| Inherent | Age | + | + | + | + | + |
|  | Sex | + |  |  |  |  |
| Immediate | Child exposures |  |  |  |  |  |
|  | Self reported illness, antibiotic use | + | + | + | + | – |
|  | Gut inflammation | | | | | |
|  | NEO, MPO, AAT |  | + | + | + | + |
|  | Enteropathogen detected | + | + | + | + | – |
|  | Micronutrient |  |  |  |  |  |
|  | Hb |  | + | + | + | + |
|  | Zn, Retinol |  | + | + | + | – |
|  | Total EBF days | + |  |  |  |  |
|  | Dietary intake, total protein |  | – | + | + | + |
| Underlying | Birth weight | + |  |  |  |  |
|  | Maternal height | + |  |  |  |  |
|  | Maternal age | + |  |  |  |  |
|  | WASH behavior |  | + | + | + | + |
|  | Improved toilet |  | + | + | + | + |
|  | Improved drinking water |  | + | + | + | + |
|  | Food security access | + |  |  |  |  |
|  | Crowding | + |  |  |  |  |
|  |  |  |  |  |  |  |
| Distant | Maternal education | + |  |  |  |  |
|  | Asset index | + |  |  |  |  |
|  | Monthly income | + |  |  |  |  |

**Supplementary table 2**: Association of aflatoxin exposure and length-for-age z-score at different time points by multiple linear regression

| Months of measurement | Coefficient | 95% Confidence Intervals | p- value |
| --- | --- | --- | --- |
| 7 months | -0.05 | -0.49, 0.39 | 0.83 |
| 15 months | 0.03 | -0.27, 0.34 | 0.83 |
| 24 months | -0.20 | -0.52, 0.11 | 0.20 |
| 36 months | -0.06 | -0.31, 0.20 | 0.67 |

All 4 models were adjusted for sex, concentrations of MPO in stool, low birth weight, maternal height, number of people sleep in one room, improved toilet, treatment of drinking water, asset categories

**Supplementary Table 3**: Association of aflatoxin exposure and stunting at different time points by multiple logistic regression

| Months of measurement | Adjusted Odds ratio | 95% Confidence Intervals | p- value |
| --- | --- | --- | --- |
| 7 months | 2.47 | 0.73, 8.36 | 0.15 |
| 15 months | 0.67 | 0.27, 1.64 | 0.38 |
| 24 months | 1.64 | 0.64, 4.21 | 0.30 |
| 36 months | 1.13 | 0.55, 2.34 | 0.74 |

All 4 models were adjusted for sex, concentrations of MPO in stool, low birth weight, maternal height, number of people sleep in one room, improved toilet, treatment of drinking water, asset categories
